# Environmental, Health, and Social Consciousness as Drivers of Organic Food Choice

**DOI:** 10.3390/nu18081242

**Published:** 2026-04-15

**Authors:** Manuel Escobar-Farfán, Iván Veas-González, Jorge Bernal-Peralta, Tiare Saavedra García, Camila Santibáñez Labraña

**Affiliations:** 1 Department of Administration, Faculty of Administration and Economics, University of Santiago of Chile, Santiago 9170020, Chile; manuel.escobar@usach.cl; 2 Departamento de Administración, Facultad de Economía y Administración, Universidad Católica del Norte, Antofagasta 1270709, Chile; tiare.saavedra@alumnos.ucn.cl (T.S.G.); camila.santibanez@alumnos.ucn.cl (C.S.L.); 3 Facultad de Administración y Economía, Universidad de Tarapacá, Arica 1000007, Chile

**Keywords:** environmental consciousness, health consciousness, social consciousness, attitude, purchase intention, organic food, PLS-SEM

## Abstract

**Background/Objectives:** The organic food market has grown substantially in recent years, yet the psychological antecedents that shape consumer attitudes and purchase intentions in emerging markets remain underexplored. This study examines how environmental, health, and social consciousness influence consumer attitudes toward organic food, and how those attitudes subsequently affect purchase intention in the Chilean context. **Methods:** Data were collected via an online survey administered to 255 Chilean consumers using non-probabilistic convenience and snowball sampling. Partial least squares structural equation modeling (PLS-SEM) was employed using SmartPLS, with bootstrapping of 5000 subsamples to test four hypothesized relationships: that environmental, health, and social consciousness each positively affect the attitude toward organic food, and that attitude, in turn, positively affects purchase intention. **Results:** All four hypotheses were supported. Social consciousness emerged as the strongest predictor of attitude (β = 0.385, *p* < 0.001), followed by environmental consciousness (β = 0.314, *p* < 0.001) and health consciousness (β = 0.165, *p* = 0.005). Attitude demonstrated a strong effect on purchase intention (β = 0.736, *p* < 0.001), explaining 54.1% of its variance. The three consciousness dimensions jointly explained 57.3% of the variance in attitude. **Conclusions:** The findings confirm that consumer attitude functions as a critical gateway through which consciousness-based motivations translate into organic food purchase intentions. Social and environmental considerations outweigh health-related concerns in driving attitudes in this context, suggesting that marketing strategies for organic food in Latin America should emphasize community and environmental values alongside individual health benefits.

## 1. Introduction

The global organic food market reached USD 134.8 billion in 2022, a nearly nine-fold increase from USD 15.2 billion in 1999 [1]. This growth reflects a structural shift in consumer preferences driven by heightened awareness of environmental degradation, food safety concerns, and health consciousness [2,3]. Despite representing only 1% of organic food sales in Latin America [4], Chile has emerged as a relevant case study: organic exports surpassed USD 311 million in 2020, covering more than 50 countries [5], while domestic consumption remains constrained by price barriers and limited availability.

Understanding what motivates consumers to purchase organic food has attracted substantial scholarly attention. The literature consistently identifies attitude as one of the most robust predictors of organic food purchase intention [6,7,8,9]. Convergent evidence from healthy food brand studies confirms this attitudinal mechanism across adjacent consumption contexts [10,11,12,13]. Yet, the upstream question, what psychological dimensions shape that attitude, has received comparatively less attention, particularly in developing-country contexts where economic constraints and cultural factors create distinct consumer dynamics [14,15,16].

Three psychological dimensions frequently emerge in the organic food literature as drivers of favorable consumer attitudes. Environmental consciousness refers to awareness of and concern for the ecological consequences of consumption decisions [17]. Health consciousness captures consumers’ propensity to engage in health-promoting behaviors and seek health-related information [18,19]. Social consciousness reflects sensitivity to community welfare and the social implications of individual purchasing choices [20]. Although each dimension has been studied independently in relation to organic food consumption, their joint influence on attitude and the subsequent effect of attitude on purchase intention have not been tested within a single integrative model in the Chilean context.

This study addresses that gap by testing a parsimonious model grounded in the Theory of Planned Behavior (TPB) [21,22]. Specifically, four hypotheses are examined. H1 proposes that environmental consciousness positively affects attitude toward organic food. H2 proposes that health consciousness positively affects attitude. H3 proposes that social consciousness positively affects attitude. H4 proposes that attitude positively affects purchase intention. The model was tested with 255 Chilean consumers using PLS-SEM, a technique well-suited to complex latent-variable models with a predictive focus [23,24].

The contribution of this study is threefold. First, it provides an integrative empirical test of three dimensions of consciousness as simultaneous antecedents of attitude in a Latin American market, extending the largely Western-centric evidence base. Second, it identifies the relative weights of each consciousness dimension, revealing that social and environmental considerations outweigh health concerns in shaping attitudes toward organic food in Chile. Third, the findings offer actionable insights for marketers and policymakers seeking to promote sustainable food consumption in emerging economies.

The remainder of the paper is organized as follows. Section 2 presents the theoretical framework and develops the four hypotheses. Section 3 describes the research methodology. Section 4 reports the measurement and structural model results. Section 5 discusses the findings and their implications. Section 6 concludes the paper.

## 2. Theoretical Framework and Hypothesis Development

The theoretical foundation of this study is the Theory of Planned Behavior (TPB), proposed by Ajzen [21]. The TPB posits that behavioral intentions are primarily determined by attitudes toward the behavior, subjective norms, and perceived behavioral control. Among these, attitude, defined as the degree to which an individual evaluates a behavior favorably or unfavorably [11], Ajzen has consistently demonstrated the strongest predictive power for organic food consumption [6,25,26]. In the present model, we focus on the attitudinal pathway and examine three psychological dimensions that theoretically precede attitude formation: environmental consciousness, health consciousness, and social consciousness. This framing is consistent with extended applications of the TPB, in which antecedent psychological constructs are proposed to shape attitude rather than to operate as independent predictors of intention [27,28].

The model focuses exclusively on the attitudinal pathway, a scope decision justified by meta-analytic evidence consistently identifying attitude as the dominant predictor of organic food purchase intention [6,29], and by research in emerging markets showing that attitude accounts for the majority of explained variance in intention, even when normative components are included [15,26]. Broader psychological orientations—environmental, health, and social consciousness—are treated as upstream determinants of attitude, consistent with the Value-Belief-Norm framework [30] and with extended TPB applications in the sustainability consumption literature [28,31].

### 2.1. Environmental Consciousness and Attitude Toward Organic Food

Environmental consciousness refers to consumers’ concern for the ecological state of their environment and their awareness of how consumption choices affect natural ecosystems [2,17]. Environmentally conscious consumers tend to perceive organic food as an ecologically responsible option because its production avoids synthetic pesticides and fertilizers, promotes biodiversity, and reduces soil degradation [32,33]. Empirical evidence across multiple cultural contexts supports this relationship. Yadav and Pathak [34] found that environmental concern was a significant predictor of positive attitudes toward organic food among Indian consumers. Zhou et al. [35] demonstrated that environmental considerations positively shaped attitudes toward organic products among Chinese consumers. More recently, Akter et al. [36] found that consumers with greater environmental concern exhibit more favorable attitudes toward organic food. Parashar et al. [31] further demonstrated that environmental awareness significantly moderates the relationship between health consciousness and organic food attitude in an Indian context. This finding additionally confirms environmental concern as a critical antecedent of attitude formation. In a European context, Ferreira and Pereira [37] showed that environmental concern had a significant positive effect on both purchasing attitude and purchase intention among Portuguese consumers of organic food. Based on the preceding theoretical and empirical evidence, the following hypothesis is proposed:

**H1.**  *Environmental consciousness has a direct and positive effect on attitude toward organic food.*

### 2.2. Health Consciousness and Attitude Toward Organic Food

Health consciousness refers to consumers’ predisposition to reflect on their health and to take proactive steps to maintain or improve it, including through dietary choices [18,38]. Health-conscious consumers tend to view organic food favorably because they perceive it as free from potentially harmful chemicals, pesticides, and artificial additives, and as nutritionally superior to conventionally produced alternatives [39,40]. This perception translates into more positive evaluations of organic food as a behavioral option.

Michaelidou and Hassan [18] identified health consciousness as one of the most influential predictors of attitudes and intentions toward organic food. Subsequent studies by Hsu et al. [41] and Prentice et al. [42] corroborated these findings in Asian contexts. McFadden and Huffman [43] showed that health-conscious consumers are willing to pay price premiums for organic products, indicating that health concerns create strong attitudinal foundations for organic food choice. However, Husic-Mehmedovic et al. [44] cautioned that health consciousness, while generating positive inclinations, does not always translate into consistent behavioral outcomes, a pattern that reinforces the importance of attitude as an intermediate mechanism. Recent evidence strengthens this position: Pan et al. [45] affirmed that consumers of health and wellness foods reported that health consciousness significantly shapes attitudes, which in turn mediate the path to purchase intention. In the same context, Baş et al. [46] corroborated this finding in a large-scale study of Turkish organic food consumers, and Bazhan et al. [47] reported health consciousness as the strongest overall predictor of organic food purchase behavior in an Iranian sample. Parashar et al. [31] additionally confirmed health consciousness as a significant antecedent of attitude in the South Asian context, a finding further corroborated in Latin American healthy food contexts [48]. Drawing on the reviewed literature, this study advances the following hypothesis:

**H2.**  *Health consciousness has a direct and positive effect on attitude toward organic food.*

### 2.3. Social Consciousness and Attitude Toward Organic Food

Social consciousness reflects consumers’ awareness of and concern for the well-being of their community and social environment, including support for local economies, equitable food systems, and collective health [20,49]. Socially conscious consumers tend to view organic food favorably because its production is associated with supporting local producers, reducing the social costs of industrial agriculture, and contributing to community sustainability [50,51].

Rana and Paul [20] identified social consciousness as a significant motivator of positive attitudes and purchase intentions for organic food, noting its role in encouraging consumers to support local and sustainable markets. Hansen et al. [19] found that social values substantially influenced organic food identity and behavioral outcomes, particularly among consumers who perceive their choices as contributing to collective welfare. Molinillo et al. [49] demonstrated in a cross-national study of Spanish and Brazilian millennials that social consciousness was positively associated with attitudes toward organic food. Su et al. [32] found that social consciousness, alongside health consciousness, significantly shaped attitudes toward organic food and subsequent purchase frequency within a TPB framework, providing direct evidence of this construct’s role in emerging market contexts. In a Latin American setting, Leyva-Hernández et al. [52] showed that consumer values with social dimensions influenced attitudes toward organic food in Mexico, with attitude mediating the path to purchase intention, a finding that is particularly relevant given the cultural proximity to the Chilean context studied here. In light of the foregoing evidence, this study proposes the following hypothesis:

**H3.**  *Social consciousness has a direct and positive effect on attitude toward organic food.*

### 2.4. Attitude and Purchase Intention Toward Organic Food

Within the TPB framework, attitude represents the primary antecedent of behavioral intention [21,53]. In the organic food literature, this relationship has been extensively validated. Çabuk et al. [7] demonstrated that attitude mediates the effects of various consumer characteristics on organic food purchase intention. Nuttavuthisit and Thøgersen [25] found that trust-based positive attitudes were critical for converting consumer interest into actual purchase intent. Scalco et al. [6] conducted a meta-analytic structural equation modeling analysis grounded in the Theory of Planned Behavior, integrating evidence from multiple independent studies across diverse cultural contexts. Their findings confirmed that attitude is the dominant predictor of organic food purchase intention, consistently outperforming subjective norms and perceived behavioral control in terms of explanatory power. This meta-analytic evidence provides cross-cultural support for centering the present model on the attitudinal pathway.

More recent evidence reinforces this pattern. Tandon et al. [26] showed that attitude accounted for a substantial share of the variance in organic food purchase intention, even after controlling for other psychological and social factors. Fleseriu et al. [54] similarly found that attitude was the most robust TPB component for predicting organic food purchase intentions among Romanian consumers. Müller-Pérez et al. [55] replicated this pattern across healthy product categories using an integrative TPB model. Also, Ham et al. [8] extended these findings to behavioral outcomes, demonstrating that the attitude-intention link is particularly strong for products with credence attributes, a category to which organic food clearly belongs.

At the meta-analytic level, Leonidou et al. [29] synthesized a large corpus of primary studies on organic food consumer behavior, identifying attitude as the dominant predictor of purchase intention across cultural contexts. Their analysis revealed that the effect size of attitude on intention consistently exceeded that of subjective norms and perceived behavioral control. Furthermore, Leonidou et al. [29] identified key boundary conditions of this relationship, including consumer knowledge and product involvement, underscoring the robustness of the attitude–intention link as a cross-cultural finding and reinforcing its centrality in the present model. In the present model, attitude is expected to function as the primary pathway through which consciousness-based motivations translate into purchase intentions. Accordingly, the following hypothesis is formulated:

**H4.**  *Attitude has a direct and positive effect on organic food purchase intention.*

**Figure 1:**
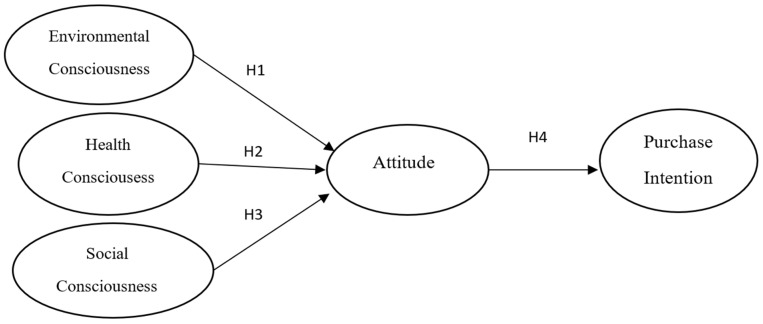
Proposed theoretical model.

The proposed model is summarized in Figure 1.

## 3. Materials and Methods

### 3.1. Research Design and Sampling

This study adopts a quantitative, cross-sectional, explanatory design. Data were collected during November 2024 through a self-administered online survey distributed via Google Forms. The target population comprised adult consumers (≥18 years) who had purchased food in Chile. The study was conducted in accordance with the Declaration of Helsinki. Ethical approval was granted by the Ethics Committee of Universidad Católica del Norte (protocol code 035, 24 August 2022). All participants provided informed consent prior to completing the survey. Sampling was non-probabilistic, combining convenience and snowball technique, an approach widely used and accepted in organic food consumer research [26,49].

The minimum sample size was determined a priori using GPower 3.1.9.7, specifying a linear multiple regression with an effect size of f^2^ = 0.10, α = 0.05, desired power of 0.95, and three predictors, yielding a minimum requirement of 254 observations (actual power = 0.951). A total of 264 responses were collected, of which 255 were valid after excluding nine respondents who did not provide informed consent or did not meet the age criterion. The final sample size satisfies both the GPower requirement and the commonly cited rule of thumb for PLS-SEM: 10 times the maximum number of arrows pointing to any construct [24].

Demographic characteristics of the sample are summarized in Table A1. The sample was slightly more male (53.3%) and young, with 59.2% of respondents aged 18–25 years. Regarding income, 44.3% of respondents reported monthly incomes below CLP 200,000, consistent with the student-heavy age distribution. These characteristics should be considered when interpreting the results and generalizing the findings.

### 3.2. Measurement Instrument

All constructs were measured using reflective scales adapted from validated instruments in the organic food literature. Items were contextualized to organic food consumption in Chile without altering their original conceptual meaning. A seven-point Likert scale was used, anchored at 1 (strongly disagree) and 7 (strongly agree). The full instrument is presented in Table A2.

Environmental consciousness (CA) was measured using six items adapted from Wijekoon and Sabri [2] and Jeyakumar et al. [56] capturing consumers’ concerns about environmental conditions, the ecological impact of food choices, and support for environmentally responsible production. Health consciousness (CPS) was measured using three items adapted from Molinillo et al. [49] to assess the extent to which consumers actively think about and prioritize their personal health. Social consciousness (CS) was measured using three items adapted from Molinillo et al. [49] that capture awareness of the community-level implications of organic food consumption. Attitude (ACT) was measured using three items adapted from Kumar et al. [16] that assessed the overall evaluation of organic food purchasing as good, favorable, and desirable. Purchase intention (IC) was measured using three items adapted from Aitken et al. [9] that reflect consumers’ stated likelihood of purchasing organic food on their next shopping trip.

Content validity was established through expert review by three academic specialists in consumer behavior and food marketing prior to data collection. A pilot test with 30 consumers confirmed item comprehensibility; no modifications were required.

### 3.3. Common Method Bias

Given the cross-sectional, single-source design, procedural and statistical measures were adopted to minimize common method bias. Procedurally, the survey guaranteed respondent anonymity to reduce evaluative apprehension [57]. Statistically, the full collinearity variance inflation factor (VIF) approach proposed by Kock [58] was used as a post hoc assessment. All VIF values were below 3.3 (maximum observed: 2.166), confirming the absence of common method bias [58].

### 3.4. Analytical Approach

Data analysis followed the two-step PLS-SEM procedure recommended by Hair et al. [24]; assessment of the measurement model, followed by evaluation of the structural model. SmartPLS 4.0 was used for all analyses. PLS-SEM was chosen because the research objective is predictive testing of theoretically driven hypotheses about relationships among latent constructs and because PLS-SEM performs well with relatively modest sample sizes and non-normal data distributions [23,24].

Measurement model evaluation examined: (1) indicator reliability (outer loadings > 0.70); (2) internal consistency reliability (Cronbach’s α > 0.70; composite reliability ρc > 0.70); (3) convergent validity (average variance extracted, AVE > 0.50); and (4) discriminant validity via the Fornell–Larcker criterion and the heterotrait–monotrait ratio of correlations (HTMT < 0.85) [24,59]. The structural model evaluation assessed path coefficients, R^2^, and model fit, with the standardized root mean square residual (SRMR) as the criterion (SRMR < 0.08). Statistical significance was determined using bootstrapping with 5000 subsamples and a two-tailed *t*-test at α = 0.05 [24].

## 4. Results

### 4.1. Measurement Model Assessment

Table 1 presents the results of the measurement model evaluation. All outer loadings exceeded the 0.70 threshold (range: 0.829–0.972), confirming the reliability of the individual indicators. Internal consistency was strong across all constructs: Cronbach’s α ranged from 0.897 to 0.956, and composite reliability (CR) ranged from 0.924 to 0.972, all well above the 0.70 benchmark. Convergent validity was supported, with AVE values ranging from 0.709 to 0.919, all exceeding the 0.50 threshold [24,59].

Discriminant validity was assessed using both the Fornell–Larcker criterion and the HTMT ratio. As shown in Table 2, the square root of each construct’s AVE (diagonal values) exceeded all inter-construct correlations, satisfying the Fornell–Larcker criterion [59]. HTMT ratios (Table 3) ranged from 0.575 to 0.769, all below the conservative 0.85 threshold [24,60], confirming discriminant validity across all construct pairs.

### 4.2. Structural Model Assessment

The structural model was evaluated after confirming adequate measurement model properties. Model fit was assessed via the SRMR, which yielded a value of 0.052 for the saturated model, well below the 0.08 threshold [61]. The estimated model SRMR was 0.083. Although marginally above the conventional threshold, this value falls within the acceptable range reported in recent PLS-SEM applications, particularly for models with higher indicator counts [24,60]. Hair et al. [24] noted that SRMR values up to 0.10 can be considered acceptable in exploratory PLS-SEM research. Multicollinearity was not a concern, as all VIF values were below 3.3 (maximum: 2.166) [58].

**Figure 2:**
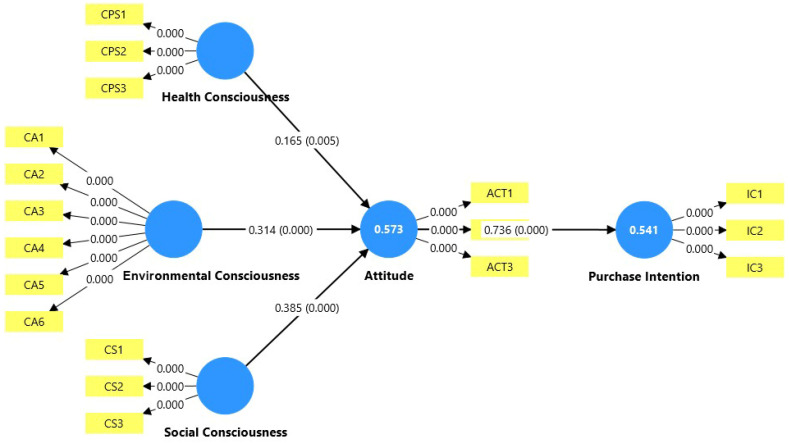
Structural model. Note: Standardized path coefficients are shown with *p*-values in parentheses. Values in bold circles represent R^2^ for endogenous constructs. CA = Environmental Consciousness; CPS = Health Consciousness; CS = Social Consciousness; ACT = Attitude; IC = Purchase Intention.

Table 4 presents the results of hypothesis testing, and the structural model is depicted in Figure 2. All four hypotheses were supported at *p* < 0.01. Social consciousness was the strongest antecedent of attitude (H3: β = 0.385, t = 4.760, *p* < 0.001), followed by environmental consciousness (H1: β = 0.314, t = 4.152, *p* < 0.001) and health consciousness (H2: β = 0.165, t = 2.781, *p* = 0.005). The three consciousness dimensions jointly explained 57.3% of the variance in attitude (R^2^ = 0.573), indicating substantial explanatory power [62]. Attitude had a strong and highly significant effect on purchase intention (H4: β = 0.736, t = 20.679, *p* < 0.001), explaining 54.1% of the variance in purchase intention (R^2^ = 0.541).

## 5. Discussion

This study examined how environmental, health, and social consciousness influence attitudes toward organic food, and how those attitudes translate into purchase intentions, among Chilean consumers. All four hypotheses were supported, providing strong empirical evidence for a TPB-grounded model in which attitudinal formation is driven by multidimensional consciousness and attitude, in turn, is the dominant driver of purchase intention.

### 5.1. The Role of Social Consciousness

Social consciousness emerged as the strongest predictor of attitude (β = 0.385), a finding that distinguishes the Chilean context from studies conducted in other markets. In Western and East Asian settings, environmental and health concerns typically dominate the formation of attitudes toward organic food [18,34,35]. The primacy of social consciousness in our sample may reflect the community-embedded character of food purchasing decisions in northern Chile, where local markets and community ties remain socially significant. This is consistent with Molinillo et al. [49], who found social consciousness to be a meaningful driver of attitudes toward organic food among Spanish millennials, and with Rana and Paul [20], who emphasized the community dimension of motivation for organic food. Su et al. [32] similarly confirmed that social consciousness is a significant predictor of attitudes toward organic food within a TPB framework, a finding that extends to other emerging markets. Particularly relevant to the present study, Leyva-Hernández et al. [52] demonstrated in a Mexican sample that social values shaped attitudes toward organic food and, through these attitudes, purchase intention, mirroring the pattern observed here in Chile and suggesting a shared Latin American consumer dynamic. The result suggests that marketing communications in the Chilean context should emphasize the community and local economy benefits of organic food consumption rather than focusing exclusively on individual environmental or health benefits.

### 5.2. The Role of Environmental Consciousness

Environmental consciousness was the second-strongest predictor of attitude (β = 0.314), consistent with the broad international evidence base [2,36]. This finding is noteworthy given Chile’s status as one of South America’s leading organic food exporters: consumers appear to be aware of the ecological dimension of organic production practices, and this awareness shapes favorable evaluations. The result aligns with Zhou et al. [35] and Akter et al. [36], confirming that environmental concern reliably predicts attitudes toward organic food even in markets where organic consumption is still emerging. Parashar et al. [31] reinforced this evidence in an Indian context, while Ferreira and Pereira [37] replicated it among Portuguese consumers, pointing to the cross-cultural robustness of this relationship. For practitioners, this suggests that environmental certification and eco-labeling strategies can effectively reinforce positive attitudes among environmentally conscious Chilean consumers.

### 5.3. The Role of Health Consciousness

Health consciousness, while a significant predictor of attitude (β = 0.165, *p* = 0.005), demonstrated the weakest effect among the three consciousness dimensions. A theoretically grounded interpretation specific to this sample connects this finding to the respondents’ income structure: 44.3% reported monthly incomes below CLP 200,000, a threshold at which organic food’s price premium represents a substantial proportional cost. Under these conditions, awareness that organic food is healthier does not translate into a proportionally stronger attitude because the behavioral option is perceived as financially constrained, reducing its evaluative salience. This mechanism is consistent with evidence from low- to middle-income emerging market contexts, where price sensitivity moderates the attitude-forming role of health consciousness [15,26,36]. Social and environmental consciousness, by contrast, operate through community and ecological values that carry no immediate economic cost signal, making them more potent attitudinal anchors in this population. Importantly, health consciousness remained statistically significant, confirming its systematic role in organic food attitude formation and corroborating the findings of Michaelidou and Hassan [18], Hsu et al. [41], Pan et al. [45], Baş et al. [46], all of which confirm health consciousness as a driver of favorable attitudes toward organic food across diverse cultural settings. That its effect is attenuated rather than absent in this sample is consistent with the findings of Husic-Mehmedovic et al. [44], who cautioned that health consciousness generates attitudinal inclinations without necessarily producing strong evaluative differentiation in all contexts.

### 5.4. The Attitude–Intention Link

The effect of attitude on purchase intention was exceptionally strong (β = 0.736, R^2^ = 0.541), reinforcing the centrality of attitude in the TPB-based literature on organic food [6,7]. The meta-analytic synthesis by Leonidou et al. [29] confirms that attitude consistently exerts the largest effect on organic food purchase intention across cultural contexts, lending strong cross-cultural support to the present finding. The magnitude of this effect in our sample is among the largest reported in recent PLS-SEM studies of organic food intention in Latin American contexts, suggesting that once a favorable attitude is formed, it translates efficiently into purchase intention in this market. This finding has important practical implications: strategies aimed at reinforcing positive attitudes, through point-of-sale information, credibility signals such as organic certification, and community-based promotion, are likely to yield strong returns in terms of stated purchase intentions.

### 5.5. Theoretical Contributions

This study makes three theoretical contributions. First, it extends the TPB framework into an integrated model in which three psychological consciousness dimensions simultaneously predict organic food attitude in an emerging Latin American market. A search of the Web of Science and Scopus databases, combining the terms ‘organic food’, ‘attitude’, ‘environmental consciousness’, ‘health consciousness’, and ‘social consciousness’, returned no prior study that tested these three antecedents jointly within a single structural model in a Latin American context. This absence confirms the originality of the present contribution, which enriches a literature predominantly shaped by Western and East Asian evidence. Second, it establishes the relative explanatory weight of each consciousness dimension, showing that social consciousness is the dominant driver in the Chilean context, a finding that challenges the assumption of health consciousness primacy implicit in much of the organic food literature. Third, the study confirms that the attitude–intention relationship remains robust across cultural contexts, supporting the generalizability of this core TPB mechanism while demonstrating that its antecedents vary by cultural setting.

### 5.6. Managerial Implications

For organic food producers and retailers operating in Chile, the findings suggest a reorientation of marketing strategy. Rather than centering communication on individual health benefits, which, while relevant, show weaker effects on attitude formation, campaigns should foreground the community and environmental dimensions of organic food choice. Messages that connect organic purchasing to support for local producers, community economic development, and environmental stewardship are likely to generate stronger attitudinal responses. For public policy, these results suggest that sustainability and community food system initiatives may be effective levers for promoting organic food consumption in contexts similar to Chile.

### 5.7. Strengths and Limitations

This study has several methodological and conceptual strengths. It provides an integrative empirical test of three dimensions of consumer consciousness as simultaneous antecedents of organic food attitude within a single structural model, a design not previously applied in the Latin American context. The use of PLS-SEM with 5000 bootstrap subsamples, rigorous measurement model evaluation, and full collinearity VIF testing for common method bias collectively ensures methodological rigor. The sample size satisfies both the a priori GPower requirement and standard PLS-SEM guidelines.

However, several limitations should be acknowledged. First, the cross-sectional design precludes causal inference; longitudinal designs would more appropriately capture attitudinal change over time. Second, the convenience sample is slightly more young (59.2% aged 18–25 years) and geographically concentrated in northern Chile, limiting generalizability to broader Latin American populations. Third, the self-administered online survey format may introduce recall bias compared to face-to-face interview methods. Fourth, actual purchase behavior was not measured, leaving the attitude–intention–behavior gap unexplored. Fifth, the model excludes subjective norms and perceived behavioral control, which are components of the full TPB framework and whose inclusion in future studies would provide a more complete account of the determinants of organic food purchase intention.

## 6. Conclusions

This study demonstrates that environmental, health, and social consciousness are significant antecedents of consumers’ attitudes toward organic food in Chile, and that these attitudes strongly predict purchase intention. Social consciousness emerged as the dominant driver of attitude, a finding that challenges the health-primacy assumption common in the organic food literature and points to a distinctively community-oriented consumer dynamic in the Latin American context. These results extend the TPB-grounded evidence base to an underrepresented regional market and offer actionable insights for practitioners: marketing strategies should foreground community and environmental values over individual health messaging. Future research should address the study’s limitations through longitudinal designs, probabilistic sampling across diverse socioeconomic and age groups, and the inclusion of actual behavioral measures, subjective norms, and perceived behavioral control, as well as replication in other Latin American markets to assess the cross-cultural generalizability of these findings.

## Figures and Tables

**Table 1:** Measurement model: factor loadings, reliability, and convergent validity.

Construct	Indicator	Loading	α	CR	AVE
Environmental Consciousness (CA)	CA1	0.875	0.897	0.924	0.709
	CA2	0.781			
	CA3	0.846			
	CA4	0.851			
	CA5	0.853			
	CA6	0.829			
Health Consciousness (CPS)	CPS1	0.960	0.953	0.970	0.914
	CPS2	0.954			
	CPS3	0.954			
Social Consciousness (CS)	CS1	0.857	0.902	0.939	0.838
	CS2	0.945			
	CS3	0.941			
Attitude (ACT)	ACT1	0.955	0.956	0.972	0.919
	ACT2	0.962			
	ACT3	0.960			
Purchase Intention (IC)	IC1	0.942	0.953	0.970	0.915
	IC2	0.973			
	IC3	0.954			

Note: α = Cronbach’s alpha; CR = composite reliability (ρc); AVE = average variance extracted.

**Table 2:** Discriminant validity: Fornell–Larcker criterion.

Construct	CA	CPS	CS	ACT	IC
Environmental Consciousness (CA)	**0.842**				
Health Consciousness (CPS)	0.639	**0.956**			
Social Consciousness (CS)	0.688	0.569	**0.915**		
Attitude (ACT)	0.714	0.558	0.698	**0.959**	
Purchase Intention (IC)	0.634	0.551	0.661	0.735	**0.956**

Note: Diagonal values (bold) represent the square root of AVE. Off-diagonal values are inter-construct correlations.

**Table 3:** Discriminant validity: HTMT ratios.

Construct	CA	CPS	CS	ACT
Health Consciousness (CPS)	0.687	—		
Social Consciousness (CS)	0.758	0.613	—	
Attitude (ACT)	0.769	0.584	0.752	—
Purchase Intention (IC)	0.682	0.578	0.712	0.768

Note: All values below the 0.85 threshold, confirming discriminant validity [24,60].

**Table 4:** Structural model results: path coefficients and hypothesis testing.

Hypothesis	Relationship	β	Mean	SD	t-Value	*p*-Value	Decision
H1	CA → ACT	0.314	0.315	0.076	4.152	<0.001	Supported
H2	CPS → ACT	0.165	0.167	0.059	2.781	0.005	Supported
H3	CS → ACT	0.385	0.384	0.081	4.760	<0.001	Supported
H4	ACT → IC	0.736	0.737	0.036	20.679	<0.001	Supported

Note: CA = Environmental Consciousness; CPS = Health Consciousness; CS = Social Consciousness; ACT = Attitude; IC = Purchase Intention; β = standardized path coefficient; SD = standard deviation. Bootstrapping with 5000 subsamples.

## Data Availability

Data can be requested by writing to the corresponding authors of this publication.

## Appendix A

**Table A1:** Demographic characteristics of the sample (n = 255).

Characteristic	Category	n	%
Gender	Female	116	45.5
	Male	136	53.3
	Prefer not to say	3	1.2
Age (years)	18–25	151	59.2
	26–35	55	21.6
	36–55	36	14.1
	56 or older	13	5.1
Monthly income (CLP)	<200,000	112	43.9
	200,001–800,000	84	32.9
	>800,000	59	23.1

**Table A2:** Measurement instrument.

Code	Item	Source
	Environmental Consciousness (CA)	
CA1	I am concerned about the conditions of our environment.	Wijekoon & Sabri [2]; Jeyakumar et al. [56]
CA2	Environmental concerns influence my food choices.	
CA3	Organic food is environmentally friendly.	
CA4	Chemical fertilizers are harmful to the environment.	
CA5	Everyone should be concerned about our environment.	
CA6	I consider the environmental impact when buying food.	
	Health Consciousness (CPS)	
CPS1	I think a lot about my health.	Molinillo et al. [49]
CPS2	I am very conscious about my health.	
CPS3	I am usually concerned about my health.	
	Social Consciousness (CS)	
CS1	I am aware that buying organic food strengthens my community’s economy.	Molinillo et al. [49]
CS2	I normally support local shops.	
CS3	I buy from local shops to help my community.	
	Attitude (ACT)	
ACT1	I like the idea of buying organic food.	Kumar et al. [16]
ACT2	Buying organic food is a good idea.	
ACT3	I have a favorable attitude toward organic food.	
	Purchase Intention (IC)	
IC1	I intend to buy at least one organic product on my next grocery trip.	Aitken et al. [9]
IC2	I plan to purchase organic products on my next grocery trip.	
IC3	I am willing to buy organic products on my next grocery trip.	
